# Morphometric characteristics of basal cell carcinoma peritumoral stroma varies among basal cell carcinoma subtypes

**DOI:** 10.1186/1471-5945-12-1

**Published:** 2012-03-09

**Authors:** Kyle Lesack, Christopher Naugler

**Affiliations:** 1 Room G503, O'Brien Centre for the BHSc, 3330 Hospital Drive N.W., Calgary, AB T2N 4N1, Canada; 2 Department of Pathology and Laboratory Medicine, University of Calgary and Calgary Laboratory Services, C414, Diagnostic and Scientific Centre, 9, 3535 Research Road NW, Calgary, AB, Canada T2L 2K8

## Abstract

**Background:**

The role that the peritumoral stroma plays in the growth of tumours is currently poorly understood. In this manuscript the morphometric characteristics of basal cell carcinoma subtypes and their associated peritumoral stromas are presented.

**Methods:**

Ninety eight digitized basal cell carcinoma histology slides were categorized as infiltrative, nodular, or superficial subtypes, and were analysed using a combination of manual and computer-assisted approaches. The morphometric characteristics of the tumour nests and their associated peritumoral stroma were quantified, and the presence of a marked immune reaction or elastosis was noted.

**Results:**

The tumour to stroma ratio was different among each tumour subtype. Elastosis was identified in a greater proportion of the infiltrative tumours.

**Conclusions:**

Quantitative differences exist between the peritumoral stroma of basal cell carcinoma subtypes. Future work exploring the relation between these morphometric differences and biochemical variations in peritumoral stroma may further our understanding of the biology of carcinoma development.

**Trial Registration:**

Not applicable.

## Background

Carcinomas (cancers derived from epithelial cells) are accompanied by a specialized peritumoral stroma which plays an important but poorly understood role in the growth and metastasis of the tumour [1-4]. The peritumoral stroma is composed largely of activated fibroblasts, inflammatory cells and vasculature and engages in a complex crosstalk with the developing tumour. This interplay appears to be mediated largely by soluble paracrine factors [1-17].

Modifications to the peritumoral stroma include alterations in the secretion of extracellular matrix proteins and growth factors by fibroblasts. Particularly important are the stromally expressed matrix metalloproteinases (MMPs), which may have wide-ranging effects on tumor growth, angiogenesis and metastasis [17]. A number of other soluble fibroblast-derived factors have been shown to induce carcinoma initiation and progression with Wnt-1, HGF and TGF-β playing particularly prominent roles [18]. Gene expression profiling of both stroma and tumor in an inducible human neoplasia model showed that during tumour growth epidermal tissue first showed increased expression of genes mediating cellular biosynthesis followed by increased expression of genes promoting proliferation and lastly increased expression of genes involved in extracellular matrix remodelling and increased cell motility. In contrast, gene expression by stromal fibroblasts shows a different pattern with early increased expression of genes involved in angiogenesis [13].

In addition to the paracrine factors secreted by fibroblasts, inflammatory cells in the peritumoral stroma also secrete growth factors, as well as reactive oxygen species which may further effect cancer growth [19]. Likewise, complex interactions involving activated fibroblasts and tumor-associated macrophages promote angiogenesis [10].

As a model for the interaction between epithelial tumor cells and stroma, basal cell carcinomas (BCC) offer a number of unique opportunities to study these processes. In addition to being the most common malignant tumor, at least in Caucasians [20], BCC are accompanied by a well-defined peritumoral stroma. The contribution of this peri-tumoral stroma to the growth of BCCs is particularly interesting as several studies have found that the growth of BCCs is stroma-dependent and limited by host immune response. For example, human to mouse mice BCC transplants are only successful in T, B, and NK cell deficient mice [21-23]. Moreover, the variety of morphological patterns of this tumor provides an opportunity to study associations between stromal variation and tumor architecture.

Previous work on BCC peritumoral stroma has defined some qualitative features of this tissue. Work by Humphreys et al. has shown an increased number of mast cells and dermal dendrocytes expressing CD34 and GP1b adjacent to BCC tumor nests, although no differences were noted among tumor subtypes [24]. In contrast, among the three most common histological patterns of BCC growth (nodular, infiltrative and superficial) striking differences in the expression of CD44 glycoprotein expression molecules have been observed, with infiltrative tumors showing the greatest expression of CD44 isoforms as revealed by immunohistochemical staining [25]. Dingemans et al. suggest that this finding indicates that CD44 may play an important role in BCC biology and specifically in determining the histological growth pattern and degree of tumor invasiveness. This same study reported that α-smooth muscle actin positivity (as a marker of myofibroblastic differentiation) tended to be greatest in infiltrative tumor areas [22]. Thus there appear to be qualitative differences in BCC peritumoral stroma which are associated with variations in histological growth pattern.

In addition to biochemical factors, some morphometric characteristics of BCCs have been explored. Studies performed by Dixon et al. [26] and Jacobs et al. [24] have investigated the relationship between tumour nest morphology and tumour aggressiveness. Significant relationships were found between non-circular nests, smaller degrees of peripheral palisading, and tumour aggressiveness. Dixon et al. [26] and Jacobs et al. [27] also found that fibrous stroma was associated with less aggressive tumours, and that hyalinization was present in the stromas of more aggressive tumours. A couple of studies have also explored the relationship between nuclear cell morphometry and tumour aggressiveness [28,29]. De Rosa et al. [28] claimed that higher nuclear areas and perimeters were associated with increased aggressiveness; however Appel et al. failed to replicate this finding [29]. Of particular note, de Rosa et al. also observed an increased incidence of hyalinization in the stroma of the aggressive tumours [28]. Considering that previous studies focused primarily on the tumour nests, further investigation into the relationships between the peritumoral stroma and tumour morphometry may be of interest.

The purpose of this study is to employ advanced image analysis techniques to look for patterns in the quantitative relationship between different BCC histology patterns and the BCC peritumoral stroma.

## Methods

### Case selection/image acquisition

Cases were selected from the routine clinical caseload of the senior author. Images of 98 hematoxylin and eosin stained slides were digitized using a commercial Aperio CS-O slide scanner at 80 × magnification, and sections containing BCC were stored as JPEG images (1072 × 902 pixels). To avoid possible confounding effects of biopsy site changes and wound healing on the morphological assessment of tumour subtype [30,31], we excluded any cases that were previously biopsied. We did not include any cases with prior biopsies. Do to a lack of recognized classification schemes for basal cell carcinoma stroma, we did not attempt to sub-classify stroma types.

The study protocol was reviewed by the Calgary Laboratory Services Research Committee and deemed to represent a method development project not requiring further ethics approval (CLS Study Code RS-11-504).

### Image analysis

Printed copies of each of the 98 BCC images were initially evaluated manually. In each BCC image five neoplastic regions of interest (ROI) were identified, and their stromal diameters and tumour radii were delineated. ROIs were selected based on the following criteria: (i) the area must be free of technical artefacts such as variations in staining and cutting of slides and must not include borders of the tissue sample or image, and (ii) ROIs must include only one subtype of tumor (i.e. not transitional or combined tumours). When possible each ROI was selected from a separate tumour nest. Multiple ROIs were identified in the same tumour nest only when the total number of tumour nests was fewer than five. The tumour radii and stroma diameters were measured directly from the printed image for each ROI. A limitation to this approach arose where tumour nests were close together and the intervening space was entirely composed of peri-tumoural stroma. In this instance stroma attributable to each nest was arbitrarily defined as the stroma between them divided by two. Finally, the presence of inflammation or elastosis was noted, and the tumour subtype for each slide was categorized as nodular, superficial, or infiltrative.

In addition to the manual evaluation, the open source digital imaging and analysis software ImageJ (version 1.44p) was used to quantify the morphology of the tumour nests in each slide. By measuring the circularity of the tumour nest associated with each of the ROIs, the tumour nest roundness was quantified. The circularity measurement in ImageJ is calculated as follows:

$$circularity\;=\;\frac{4\;\cdot \;\pi \;\cdot \;area}{perimeter^{2}}$$

A circularity measurement of 1 indicates a perfect circle, while more elongated shapes result in lower values [32]. In order to avoid artificially low circularity measurements, tumour nests cut off extensively by the image edge were excluded from this analysis.

### Visual tumour classification

Images were classified into tumor subtypes according to the definitions provided by Crowson [33]. The 98 images were composed of 23 superficial BCCs, 60 nodular BCCs and 15 infiltrative BCCs. The presence of both solar elastosis and an accompanying chronic immune response were subjectively scored with absent and mild counted as negative and moderate and marked counted as present.

### Statistical analysis

Data analyses were performed using IBM SPSS Statistics v19. Box plots of the morphometric statistics and bar plots of the categorical statistics were created using R v2.13.0. Non-parametric tests were employed, as initial calculations indicated non-normal distributions. An analysis of variance was performed using the Kruskal-Wallis test, and Mann-Whitney tests were employed when significant differences between the groups were found. Categorical variables were analysed using the Chi-squared test. Interaction effects between continuous and categorical variables were tested using binary logistic regression.

## Results

**Figure 1:**
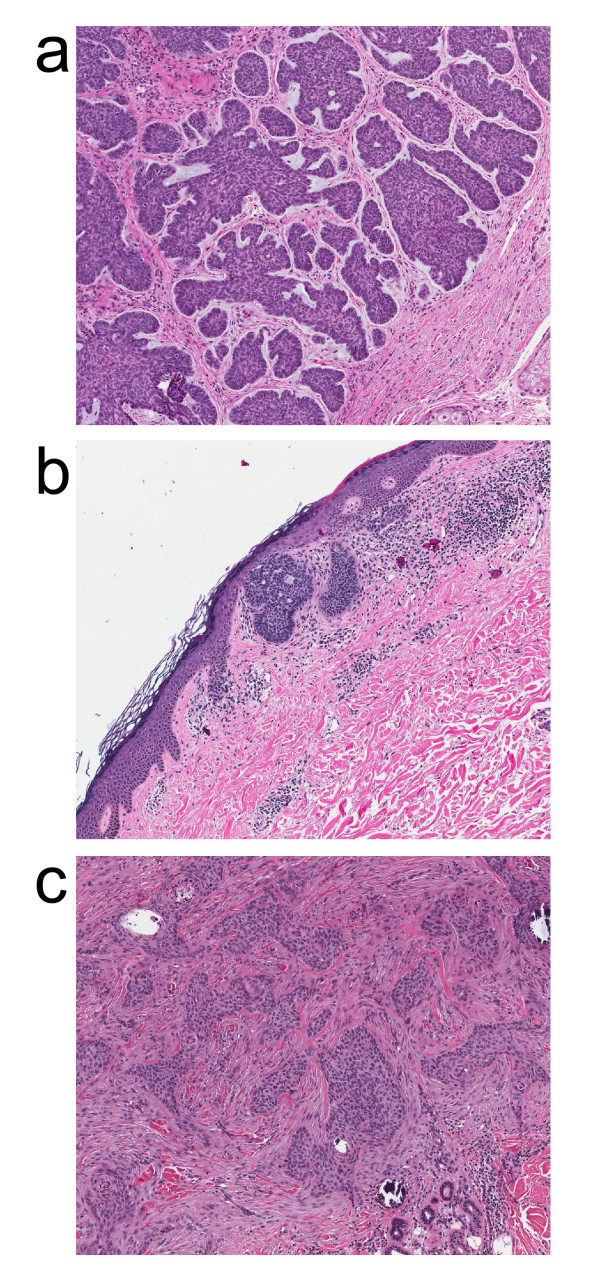
**Examples of basal cell carcinoma subtypes included in this study: (a) nodular, (b) superficial, (c) infiltrative**.

Examples of the different basal cell carcinoma subtypes included in this study are illustrated in Figure 1. Statistically significant differences were found among tumor subtypes with regard to median tumour radii (p = 0.005), stroma diameters (p = 0.008), and tumour to stroma ratio (p = 0.000006). Post hoc pairwise comparisons showed different median tumour radii (p = 0.0009) and stroma diameters (p = 0.003) between the infiltrative and nodular subtypes. For each of the tumour subtypes different tumour-to-stroma ratios were obtained (infiltrative-nodular p = 0.00006, infiltrative-superficial p = 0.006, nodular-superficial p = 0.005). There was insufficient statistical evidence to conclude that circularity varied by tumour subtype (p-value = 0.3). The morphometric comparisons are summarized in Figure 1 and Table 1.

**Figure 2:**
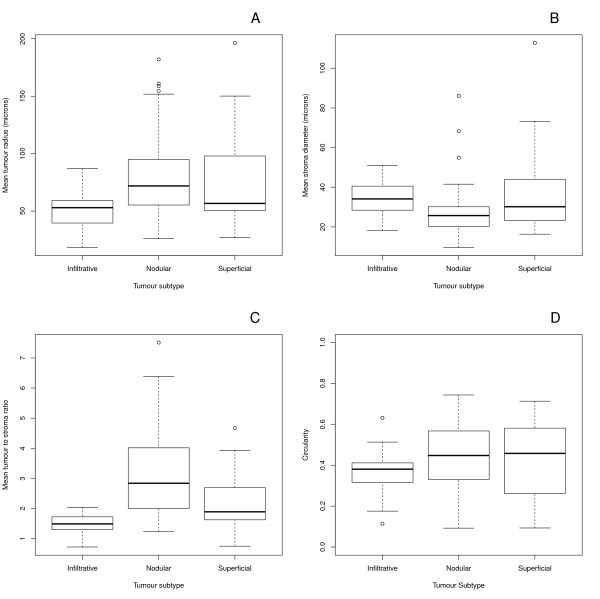
**Box and whisker plots for the mean tumour radii (A), stroma diameters (B), tumour to stroma ratios (C), and circularities by tumour subtype (D)**.

**Table 1:** Morphometric characteristics of basal cell carcinomas

Variable	Infiltrative (n = 15)	Nodular (n = 60)	Superficial (n = 23)	All (n = 98)	*P*1 (Infiltrative-Nodular)	*P*2 (Infiltrative-Superficial)	*P*3 (Nodular-Superficial)
Tumour radius (microns)--median (IQR)	52.92 (39.56, 59.22)	71.83 (55.45, 94.95)	56.62 (50.32, 97.92)	64.29 (53.16, 91.24)	**0.0009**	0.06	0.3

Stroma width (microns)--median (IQR)	34.12 (28.44, 40.56)	25.72 (20.28, 30.17)	30.17 (23.24, 44.02)	27.44 (21.52, 34.86)	**0.003**	0.5	0.08

Tumour to stroma ratio--median (IQR)	1.492 (1.308, 1.726)	2.842 (2.023, 4.005)	1.894 (1.627, 2.691)	2.170 (1.692, 3.330)	**1.129e-06**	**0.006**	**0.005**

Circularity--median (IQR)	0.3808 (0.3158, 0.4112)	0.4475 (0.3328, 0.5672)	0.4580 (0.2616, 0.5813)	0.4266 (0.3155, 0.5652)	0.1	0.3	0.97

*P*1 = p-value obtained for the difference between the median variable values between the infiltrative and nodular subtypes. A two tailed Mann-Whitney test was used with a 95% confidence interval

*P*2 = p-value obtained for the difference between the median variable values between the infiltrative and superficial subtypes. A two tailed Mann-Whitney test was used with a 95% confidence interval

*P*3 = p-value obtained for the difference between the median variable values between the nodular and superficial subtypes. A two tailed Mann-Whitney test was used with a 95% confidence interval

IQR = interquartile range

The presence of elastosis was correlated with the tumour subtype (p = 0.033), as eight of the fifteen (53%) infiltrative, twelve of the sixty (20%) nodular, and six of the twenty-three (26%) superficial tumours contained elastosis. The presence of an immune reaction was not found to be significant (p = 0.1). The categorical statistics are summarized in Table 2. Possible interaction effects between dermal elastosis (significant in univariate analysis) and the continuous variables were explored using binary logistic regression. No statistically significant associations were found using this approach (analysis not shown).

**Table 2:** Presence of immune reactions or elastosis in basal cell carcinomas.

Characteristic	Infiltrative^1^ (n = 15)	Nodular^1^ (n = 60)	Superificial^1^ (n = 23)	*P*1 (Infiltrative-Nodular)	*P*2 (Infiltrative-Superficial)	*P*3 (Nodular-Superficial)
Immune Reaction	4 (0.27)	18 (0.30)	12 (0.52)	1.0	0.32	0.14

Elastosis	8 (0.53)	12 (0.20)	6 (0.26)	**0.0057**	0.17	0.37

Chi-squared tests were used except where an individual cell had a value less than five in which case a Fisher's exact test was used

^1 ^Data are presented as totals and proportions

*P*1 = p-value obtained for the difference between the median variable values between the infiltrative and nodular subtypes

*P*2 = p-value obtained for the difference between the median variable values between the infiltrative and superficial subtypes

*P*3 = p-value obtained for the difference between the median variable values between the nodular and superficial subtypes

## Discussion

This study quantified the morphometric relationships between basal cell carcinoma subtypes and their peritumoral stroma. Manual and computer-assisted methods were employed to quantify size and shape statistics for infiltrative, nodular, and superficial BCC subtypes. The presence of inflammation and elastosis was also noted for each subtype.

Significant differences were found between the median tumour radii and stroma diameters of the infiltrative and nodular subtypes. The infiltrative subtype was associated with thicker stroma, but smaller tumour radii, with a correspondingly smaller tumour-to- stroma ratio. Therefore the biochemical differences previously described for infiltrative versus other BCC subtypes may be accompanied by quantitative changes in the peri-tumoral stroma.

Another noteworthy finding was that elastosis was present in a significantly higher proportion of the infiltrative subtype images. One possible explanation for this finding is that the presence of extensive sun damage contributes to the phenotype of BCC, predisposing to an infiltrative growth pattern. Possible mechanisms for such a relationship are speculative but may include differences in diffusion characteristics or paracrine factor expression in sun damaged skin. Similar arguments could be made for the presence of a chronic immune infiltrate associated with infiltrative BCC: lymphocytes could be recruited in response to some characteristic of the tumor or they may themselves secrete factors that influence the BCC phenotype.

Unexpectedly, the circularity of the tumour nest was not found to influence the tumour subtype. This may have resulted from the presence of nodular tumour nests within some of the infiltrating subtype slides, resulting in higher mean and more variable circularity measurements. Alternatively, variance of the circularity measurements among tumor nests on the same slide may provide a means of distinguishing the tumour subtypes based on their shape. Moreover, the use of different shape metrics may provide a superior means of distinguishing tumour subtypes based on their morphology. These questions could be explored in future studies.

This study was unique in several ways. First of all, a greater emphasis was placed on the relationship of the tumour morphometry and its stroma. Previous studies have focused largely on the tumour itself, therefore ignoring an important factor in tumour growth. Another benefit was that the shape statistics were quantified using an open-source digital imaging and analysis program. This facilitates replication, as ImageJ is freely available and is capable of providing circularity measurements with the default package. A further benefit of using software to quantify the tumour nest shape is that it eliminates the subjectivity resulting from categorizing the tumour shape qualitatively. Further research could improve upon this study in a couple of ways. One limitation was that rarer BCC subtypes were not included. Also, as mentioned earlier, inherent in an observational study is the fact that it was not possible to determine whether stromal differences influenced the tumour architecture or if they resulted from the architecture itself. Another limitation of this study concerns the applicability of our findings to other malignancies. Basal cell carcinomas differ from many other malignancies in that they exhibit limited metastatic potential and often lack features common to other malignancies such as aneuploidy [34].

Previous studies that have quantified the morphometry of BCC have focused on the relationship of the tumour's characteristics and its aggressiveness. Furthermore, these studies have focused mainly on the tumour, rather than its relationship to its stroma. As a result, it is not possible to directly compare the findings presented here with those of the previous BCC morphometry studies. That aside, previous authors have found that aggressive tumours have different morphometric characteristics than non-aggressive ones. This may be related to the different morphology found between the different subtypes in this study. The results obtained are also consistent with the presence of biochemical differences that were previously reported. As discussed, Humphreys et al. [24] found that the expression of CD44 glycoprotein differed amongst the infiltrative, nodular, and superficial subtypes, with infiltrative tumours showing the highest expression. The expression of CD44 may have contributed to the greater stroma diameters and lower tumour radii measured for the infiltrative subtypes in our study. Humphreys et al. [24] also reported greater α-smooth muscle actin positivity in the infiltrative tumours, and Papanikolaou [35] demonstrated integrin-linked kinase (ILK) overexpression in infiltrative tumours, which may also have contributed to the morphological differences.

## Conclusions

In this study we demonstrated that there are quantitative differences in the peritumoral stroma among BCC subtypes. Future work should focus on integrating these morphometric differences with the previously described biochemical differences in stromal microenvironments among BCC subtypes.

## Competing interests

The authors declare that they have no competing interests.

## Authors' contributions

CN devised the original study design. KL obtained the morphometric measurements. Both authors participated in drafting the manuscript. Both authors have read and approved the final manuscript.

## Pre-publication history

The pre-publication history for this paper can be accessed here:

http://www.biomedcentral.com/1471-5945/12/1/prepub

## References

[B1] Folkman J Shing Y Angiogenesis J Biol Chem 1992 267 10931 10934 1375931

[B2] Kalluri R Zeisberg M Fibroblasts in cancer Nat Rev Cancer 2006 6 392 401 10.1038/nrc1877 16572188

[B3] Tlsty TD Coussens LM Tumor stroma and regulation of cancer development Annu Rev Pathol 2006 1 119 150 10.1146/annurev.pathol.1.110304.100224 18039110

[B4] Bhowmick NA Moses HL Tumor-stroma interactions Curr Opin Genet Dev 2005 15 97 101 10.1016/j.gde.2004.12.003 15661539 PMC2819733

[B5] Tang Y Kesavan P Nakada MT Yan L Tumor-stroma interaction: positive feedback regulation of extracellular matrix metalloproteinase inducer (EMMPRIN) expression and matrix metalloproteinase-dependent generation of soluble EMMPRIN Mol Cancer Rev 2004 2 73 80 14985463

[B6] Saijo Y Tanaka M Miki M Usui K Suzuki T Maemondo M Hong X Tazawa R Kikuchi T Matsushima K Nukiwa T Proinflammatory cytokine IL-1 beta promotes tumor growth of Lewis lung carcinoma by induction of angiogenic factors: in vivo analysis of tumor-stromal interaction J Immunol 2002 169 469 475 12077278 10.4049/jimmunol.169.1.469

[B7] Samoszuk M Tan J Chorn G Clonogenic growth of human breast cancer cells co-cultured in direct contact with serum-activated fibroblasts Breast Cancer Res 2005 7 R274 R283 10.1186/bcr995 15987422 PMC1143574

[B8] Yashiro M Ikeda K Tendo M Ishikawa T Hirakawa K Effect of organ-specific fibroblasts on proliferation and differentiation of breast cancer cells Breast Cancer Res Treat 2005 90 307 313 10.1007/s10549-004-5364-z 15830145

[B9] Sironen RK Tammi M Tammi R Auvinen PK Anttila M Kosma VM Hyaluronan in human malignancies Exp Cell Res 2011 317 383 391 10.1016/j.yexcr.2010.11.017 21134368

[B10] Kitadai Y Cancer-stromal cell interaction and tumor angiogenesis in gastric cancer Cancer Microenviron 2010 3 109 116 10.1007/s12307-009-0032-9 PMC2970808 20020278

[B11] Whiteside TL The tumor microenvironment and its role in promoting tumor growth Oncogene 2008 27 5904 5912 10.1038/onc.2008.271 18836471 PMC3689267

[B12] Mueller MM Fusenig NE Friends or foes--bipolar effects of the tumour stroma in cancer Nat Rev Cancer 2004 4 839 849 10.1038/nrc1477 15516957

[B13] Reuter JA Ortiz-Urda S Kretz M Garcia J Scholl FA Pasmooij AM Cassarino D Chang HY Khavari PA Modeling inducible human tissue neoplasia identifies an extracellular matrix interaction network involved in cancer progression Cancer Cell 2009 15 477 488 10.1016/j.ccr.2009.04.002 19477427 PMC3050547

[B14] Littlepage LE Egeblad M Werb Z Coevolution of cancer and stromal cellular responses Cancer Cell 2005 7 499 500 10.1016/j.ccr.2005.05.019 15950897

[B15] Wadlow RC Wittner BS Finley SA Bergquist H Upadhyay R Finn S Loda M Mahmood U Ramaswamy S Systems-level modeling of cancer-fibroblast interaction PLoS One 2009 4 e6888 10.1371/journal.pone.0006888 19727395 PMC2731225

[B16] Ichise T Adachi S Ohishi M Ikawa M Okabe M Iwamoto R Mekada E Humanized gene replacement in mice reveals the contribution of cancer stroma-derived HB-EGF to tumor growth Cell Struct Funct 2010 35 3 13 10.1247/csf.09025 20190463

[B17] Lynch CC Matrisian LM Matrix metaloproteinases in tumor-host cell communication Differentiation 2002 2 161 174 10.1046/j.1432-0436.2002.700909.x 12492497

[B18] Bhowmick NA Neilson EG Moses HL Stromal fibroblasts in cancer initiation and progression Nature 2004 432 332 337 10.1038/nature03096 15549095 PMC3050735

[B19] Coussens LM Werb Z Inflammation and cancer Nature 2002 420 860 867 10.1038/nature01322 12490959 PMC2803035

[B20] MacKie RM Champion RH, Burton JL, Ebling FJG Epidermal skin tumours Textbook of Dermatology 1992 2 5 Oxford: Blackwell Scientific Publications 1488 1495

[B21] Hales SA Stamp G Evans M Fleming KA Identification of the origin of cells in human basal cell carcinoma xenografts in mice using in situ hybridization Br J Dermatol 1989 120 351 357 10.1111/j.1365-2133.1989.tb04159.x 2713256

[B22] Carlson JA Combates NJ Stenn KS Prouty SM Anaplastic neoplasms arising from basal cellcarcinoma xenotransplants into SCID-beige mice J Cutan Pathol 2002 29 268 278 10.1034/j.1600-0560.2002.290502.x 12100626

[B23] Kaur P Mulvaney M Carlson JA Basal cell carcinoma progression correlates with host immune response and stromal alterations: a histologic analysis Am J Dermatopathol 2006 28 293 307 10.1097/00000372-200608000-00002 16871032

[B24] Humphreys TR Monteiro MR Murphy GF Mast cells and dendritic cells in basal cell carcinoma stroma Dermatol Surg 2000 26 200 203 10.1046/j.1524-4725.2000.09207.x 10759793

[B25] Dingemans KP Ramkema MD Koopman G Van Der Wal AC Das PK Pals ST The expression of CD44 glycoprotein adhesion molecules in basal cell carcinomas is related to growth pattern and invasiveness Br J Dermatol 1999 140 17 2524 10.1046/j.1365-2133.1999.02602.x 10215763

[B26] Dixon AY Lee SH McGregor DH Factors predictive of recurrence of basal cell carcinoma Am J Dermatopathol 1989 11 222 10.1097/00000372-198906000-00005 2729527

[B27] Jacobs GH Rippey JJ Altini M Prediction of aggressive behavior in basal cell carcinoma Cancer 1982 49 533 537 10.1002/1097-0142(19820201)49:3<533::AID-CNCR2820490322>3.0.CO;2-O 7059912

[B28] De Rosa G Vetrani A Zeppa P Zabatta A Barra E Gentile R Fulciniti F Troncone G di Benedetto G Palombini L Comparative morphometric analysis of aggressive and ordinary basal cell carcinoma of the skin Cancer 1990 65 544 549 10.1002/1097-0142(19900201)65:3<544::AID-CNCR2820650327>3.0.CO;2-O 2297645

[B29] Appel T Bierhoff E Appel K Lindern JV Bergé S Niederhagen B Predictive variables for the biological behaviour of basal cell carcinoma of the face: relevance of morphometry of the nuclei Br J Oral Maxillofac Surg 2003 41 147 150 10.1016/S0266-4356(03)00074-3 12804537

[B30] Swetter SM Yaghmai D Egbert BM Infiltrative basal cell carcinoma occurring in sites of biopsy-proven nodular basal cell carcinoma J Cutan Pathol 1998 25 420 425 10.1111/j.1600-0560.1998.tb01768.x 9826167

[B31] Swetter SM Boldrick JC Pierre P Wong P Egbert BM Effects of biopsy-induced wound healing on residual basal cell and squamous cell carcinomas: rate of tumor regression in excisional specimens J Cutan Pathol 2003 30 139 146 10.1034/j.1600-0560.2003.000002.x 12641794

[B32] Ritter N Cooper J New resolution independent measures of circularity J Mathematical Imaging and Vision 2009 35 117 127 10.1007/s10851-009-0158-x

[B33] Crowson AN Crowson AN, Magro CM, Mihm MC Basal Cell Carcinoma: Clinical features, Histology, and Biology Biopsy Interpretation of the Skin 2009 Wolters Kluwer: Philadelphia 204 210

[B34] Janisson-Dargaud D Drurlach A Lorenzato M Grange F Bernard P Birembaut P Aneuploidy, but not Ki-67 or EGFR expression, is associated with recurrances in basal cell carcinoma J Cutan Pathol 2008 35 916 921 10.1111/j.1600-0560.2007.00935.x 18537864

[B35] Papanikolaou S Bravou V Gyftopoulos K Nakas D Repanti M Papadaki H ILK expression in human basal cell carcinoma correlates with epithelial-mesenchymal transition markers and tumour invasion Histopathology 2010 56 799 809 10.1111/j.1365-2559.2010.03556.x 20546345

